# Emergence of a Novel Porcine Reproductive and Respiratory Syndrome Virus 2 Strain Recombined from Two Modified Live Virus-like Strains and Its Pathogenicity for Piglets

**DOI:** 10.3390/ani16121903

**Published:** 2026-06-19

**Authors:** Yiwen Pei, Xue Gao, Shuo Feng, Danjiao Yang, Runmin Kang, Jifeng Yu, Jie Liu, Yi Qing, Zhidong Zhang, Long Zhou

**Affiliations:** 1 Key Laboratory of Animal Medicine of Sichuan Education Department, College of Animal Science and Veterinary Medicine, Southwest Minzu University, Chengdu 610041, China; 19981253316@163.com (Y.P.); gaoxue3201@163.com (X.G.); fengshuo0418@163.com (S.F.); jieliuhzau@163.com (J.L.); zhangzhidong@swun.edu.cn (Z.Z.); 2 Institute of Animal Science of Ganzi Tibetan Autonomous Prefecture of Sichuan Province, Kangding 626000, China; kangyang2468@126.com; 3 Sichuan Animal Science Academy, Sichuan Provincial Key Laboratory of Animal Breeding and Genetics, Chengdu 610066, China; angelina_0708@hotmail.com (R.K.); yujifeng2009@sohu.com (J.Y.); 4 Key Laboratory of Ministry of Education and Sichuan Province for Qinghai-Tibetan Plateau Animal Genetic Resource Reservation and Utilization, Chengdu 610041, China; 5 Chengdu Livestock and Poultry Genetic Resources Protection Center, Chengdu 610081, China

**Keywords:** porcine reproductive and respiratory syndrome (PRRS), recombination, modified live virus, molecular characteristics, phylogenetic analysis

## Abstract

Porcine reproductive and respiratory syndrome (PRRS), caused by PRRS virus (PRRSV), poses a serious threat to the global swine industry. Although modified live virus (MLV) vaccines have been widely used in the field for PRRS prevention for decades, the safety and efficacy of these vaccines have long been controversial. Here, we report a rare recombination pattern in China: the emergence of a novel NADC30-like PRRSV strain recombined from two MLV-like strains. Genome comparative analysis reveals that the SCMS2025 isolate has a non-continuous 136-amino acid deletion in the NSP2 protein and shares the highest nucleotide identity of 87.6% with lineage 5 (L5) strains. Phylogenetic analysis showed that SCMS2025 was classified into L1 (NADC30-like) strains based on ORF5 genotyping, whereas it belonged to a single branch between L1 and L5 strains based on the complete genomic sequences. Strikingly, genomic recombination analysis revealed that the newly emerged PRRSV isolate likely resulted from complex recombination events between NADC30-like and two MLV-like strains (RespPRRS MLV and TJbd14-1 MLV-like strains). Furthermore, SCMS2025 infection caused transient overt clinical signs followed by rapid recovery, indicating that the novel PRRSV isolate is a low pathogenic strain. Notably, all SCMS2025-inoculated piglets remained seronegative for PRRSV-specific antibodies throughout the entire 14-day observation period, suggesting a delayed onset of the host humoral immune response. Our study provides evidence for the ongoing evolution of PRRSV through inter lineage recombination and highlights the urgent need for safe and effective vaccines.

## Simple Summary

Porcine reproductive and respiratory syndrome (PRRS) has posed a serious threat to the global swine industry for several decades. Although modified live virus (MLV) vaccines have been widely used in the field, the safety and efficacy of these vaccines have long been a controversial issue. Here, we isolated a novel NADC30-like PRRS virus (PRRSV), designated SCMS2025, from a pig farm that practiced MLV vaccination in Sichuan province in China. Genomic sequence analysis revealed that multiple amino acid deletions occur within its genome, and the newly emerged PRRSV isolate likely originated from multiple recombination events among an NADC30-like strain and two commercial MLV-like strains (RespPRRS MLV and TJbd14-1 MLV-like strains). Furthermore, SCMS2025 infection caused transient overt clinical signs followed by rapid recovery, indicating that the SCMS2025 isolate is a low pathogenic strain. Notably, all SCMS2025-inoculated piglets remained seronegative for PRRSV throughout the experimental period. In summary, we identified a novel NADC30-like PRRSV strain representing a rare recombination pattern in China, which involved two MLV-like strains as parental donors, and evaluated its pathogenicity in piglets. Our study highlights the urgent need for safe and effective vaccines.

## 1. Introduction

Porcine reproductive and respiratory syndrome virus (PRRSV), a major causative agent of reproductive failure in breeding sows and respiratory illness in growing pigs, is responsible for substantial economic losses in the swine industry worldwide [1]. As an enveloped virus, PRRSV belongs to the order *Nidovirales*, family *Arteriviridae*, and genus *Betaarterivirus*. The genome of PRRSV is classified into two separate genotypes based on genetic diversity, PRRSV-1 (*Betaarterivirus suid 1*) and PRRSV-2 (*Betaarterivirus suid 2*), which share approximately 60% nucleotide similarity at the genome level [2].

The genome of PRRSV is a single-stranded, linear, non-segmental, positive-sense RNA, with a size of 15.0~15.4 kb [3]. The virus genome contains at least 11 open reading frames (ORFs), including ORF1a, ORF1b, ORF2a, ORF2b, ORF3–7, ORF5a, and a short transframe (TF) ORF, flanked by 5′ and 3′ non-coding regions [4]. PRRSV has eight structural proteins, GP2, E, GP3, GP4, GP5, GP5a, M, and N, which together form the basic structure of the virus. Sixteen non-structural proteins (nsp1α, nsp1β, nsp2–6, nsp2TF, nsp2N, nsp7α, nsp7β, and nsp8–12) encoded by ORF1a/1b are essential for viral replication and immune evasion [5]. PRRSV genomes often undergo mutation and recombination events, leading to the continuous emergence of novel and variant PRRSV strains worldwide; these strains have evolved into various devastating viruses that have caused immense economic losses in the global swine industry [6,7,8].

To control PRRSV infection, two types of commercial vaccines, including inactivated and modified live virus (MLV) vaccines, have been extensively applied in many countries. In China, multiple MLV vaccines, including Ingelvac PRRS MLV, JXA1-R, TJM-F92, HuN4-F112, R98, and GDr180 MLVs, have been extensively utilized in clinical practice for decades [9,10,11]. Studies have shown that the vaccination with PRRSV MLV contributes to reducing the incidence and severity of clinical diseases and the improvement in the growth performance of pig herds; however, the viral evolution, virulence reversion, and recombination of vaccine viruses have been documented [12]. At present, multiple lines of evidence support the frequent emergence of MLV-evolved and recombinant strains in the field. For example, HeN1201 and HeN1502 are MLV-like strains derived from the MLV HuN4-F112, while TJbd14-1 is classified as an MLV-like strain originating from TJM-F92 [13]. Recombination events between circulating PRRSV field strains (NADC30-like/JXA1-like strains) and a single MLV strain has been constantly reported [14,15]. However, recombination involving genomic fragments from two distinct MLV strains remains exceptionally rare. The emergence of such multi-recombinant strains is of remarkable biological and epidemiological importance.

In the present study, we isolated an NADC30-like *Betaarterivirus suid 2* strain, SCMS2025, from a pig farm practicing MLV vaccination in Sichuan province in China. The novel PRRSV isolate is likely to have undergone complex recombination events from two different MLV-like strains, which has never been described before. Furthermore, we evaluated the pathogenicity of the newly emerged SCMS2025 isolate in piglets.

## 2. Materials and Methods

### 2.1. Pig Farm Information and Sample Collection

In January 2025, a suspected clinical outbreak of PRRS occurred on a pig farm with a rearing scale of ~500 pigs in Meishan, China. The affected nursery pig herd showed fever (39.8–40.9 °C), overt signs of respiratory illness, and slight diarrhea, with approximately 25% morbidity. The pig producer started to apply a commercially available HP-PRRSV-derived MLV vaccine TJM-F92 (Sinovet, Tianjing, China) in whole herds during 2020–2022 and subsequently switched to a different MLV vaccine RespPRRS MLV (Boehringer Ingelheim, Ingelheim am Rhein, Germany) since May 2023. The farm has been operating as a closed production system, with no introduction of new pigs. The MLV vaccination has triggered PRRS instability on this pig farm, manifesting as sporadic abortions in sows along with respiratory disease in growing pigs. Ten whole blood samples collected from sick pigs were confirmed to be positive for PRRSV by reverse transcription polymerase chain reaction (RT-PCR).

### 2.2. Viral Isolation and Genomic Sequencing

All PRRSV-positive samples were tested by RT-PCR for the ORF5 regions of PRRSV. To isolate the virus, the 200 µL of PRRSV-positive serum samples were inoculated into pulmonary alveolar macrophage (PAM) cells cultured at 37 °C in a humidified 5% CO_2_ atmosphere [16]. PAM cells were prepared as follows. Briefly, bronchoalveolar lavage was performed using lung tissues collected from a 4-week-old healthy piglet originating from a farm in Sichuan province. The harvested lavage fluid was centrifuged, and the resulting cell pellet was resuspended in RPMI 1640 medium (VivaCell, Shanghai, China) containing 10% fetal bovine serum. All prepared cells were tested for PCV, PRRSV, and *Mycoplasma hyopneumoniae* via PCR or RT-PCR. Cell isolation and maintenance were carried out following a previously described protocol, using the same RPMI 1640 medium supplemented with 10% fetal bovine serum [17]. The supernatant after the seventh passage was identified by immunofluorescence assay (IFA) using a monoclonal antibody specific for PRRSV N protein (GeneTex Inc., Irvine, CA, USA) [18]. Thirteen primer pairs were used to amplify the complete genome of PRRSV (Supplementary Table S1). The PCR amplicons were sent to Sangon Biotech (Shanghai, China) for Sanger sequencing, and the full-length genomic sequence was assembled using the SeqMan module of Lasergene software (DNAStar, Madison, WI, USA). The TCID_50_ of the PRRSV isolate was determined using the method of Muench and Reed.

### 2.3. Genome Comparison and Phylogenetic Analysis

The obtained genomic sequences were analyzed using MEGA-X (v10.1.8) software and compared with representative PRRSV sequences available in GenBank [19]. Multiple sequence alignment of PRRSV strains and amino acid mutation analysis were performed using the MegAlign program included in the Lasergene software v7.1.0 (DNASTAR, Madison, WI, USA). The rooted phylogenetic trees were generated by the Neighbor-joining (NJ) method using MEGA-X (v10.1.8) software with the nucleotide sequences of PRRSV-2. The unrooted phylogenetic tree was constructed by the NJ method using the online iTOL v7 server (https://itol.embl.de/, accessed on 16 June 2026). Bootstrap values were calculated for 1000 replicates. The classification of the global PRRSV-2 strains was based on the latest classification system proposed by Yim-im [20].

### 2.4. Recombinant Analysis

Recombination analysis was performed using SimPlot software (v3.5.1) with the following parameters: Window (200 bp), Step (20 bp), Reps (100), and Kimura (2-parameter). The recombination events in SCMS2025 isolate’s full-length genomic sequences were further determined using the Recombinant Detection Program (RDP4, v4.96) with the following default parameters: sequences were set to linear, number of the permutations was set to 100, and the highest acceptable *p*-value was set to 0.05. Only recombination events detected by at least five of the seven methods (RDP, GENECONV, Bootscan, MaxChi, Chimera, 3Seq, and SiScan) were considered [14,21].

### 2.5. Experimental Infection of Animals

Ten healthy weaned Yorkshire piglets (mean age 28 ± 1 days old and mean weight 5.3 ± 0.1 kg) were purchased from a pig farm in Sichuan province with no PRRSV infection. Additionally, RT-PCR tests confirmed that the samples were negative for several common viruses, including PRRSV, classical swine fever virus (CSFV), porcine epidemic diarrhea virus (PEDV), pseudorabies virus (PRV), and porcine circovirus type 2 (PCV2). All piglets were male and underwent a 7-day acclimatization period before PRRSV challenge. Animals were randomly allocated into two experimental groups and raised in two separate environmentally controlled rooms. The rooms were temperature-controlled (25–28 °C) with appropriate ventilation and lighting. During the entire experimental period, piglets were fed with standard commercial feed and provided free access to clean drinking water. The piglets in the infected group (*n* = 5) were intranasally inoculated with 3 mL of the SCMS2025 (the seventh passage in PAMs) isolate at a titer of 1 × 10^5.0^ TCID_50_/mL. An equal volume of uninfected RPMI-1640 medium was intranasally administered to piglets in the control group (*n* = 5). All experimental procedures involving animals were reviewed and approved by the Animal Ethics Committee affiliated with the College of Animal Science and Veterinary Medicine, Southwest Minzu University (Permission Number: SMU-202501065).

Pigs were monitored daily for behavior and mental status. The body weight of each pig was recorded weekly. On days 0, 3, 6, 9, 12, and 14 post-inoculation (dpi), blood was collected from each pig regularly. All the pigs were euthanized 14 dpi with an intravenous overdose of sodium pentobarbital (Sigma-Alrdich, St. Louis, MO, USA, 100 mg/kg). Death was confirmed by the absence of a palpable heartbeat and respiratory arrest for at least 5 min. In addition, lungs, tonsils, and other tissues collected post-mortem were fixed in 4% paraformaldehyde and sent for pathological and immunohistochemical (IHC) examination [22]. All samples were processed in duplicate. One section was stained with hematoxylin and eosin (H&E) to observe microscopic lesions, and interstitial pneumonia was scored on a scale of 0 to 4 to evaluate lesion distribution and severity. Pathological severity was scored using a 0–4 scale: normal = 0, mild = 1, moderate = 2, marked = 3, and severe = 4. The other section was incubated with a PRRSV M protein-specific monoclonal antibody (GeneTex, Inc., Irvine, CA, USA) at a dilution of 1:200 for IHC staining.

### 2.6. Viral Detection by Quantitative RT-PCR (RT-qPCR)

The viral load in samples was determined by RT-qPCR using specific primers, as previously described [23]. All samples were preliminarily processed, followed by viral RNA extraction using TRizol reagent (Invitrogen, Carlsbad, CA, USA). Reverse transcription (RT) was performed using a reverse transcription kit (TOYOBO, Shanghai, China). And cDNA was then obtained from these samples. The viral load in tissues and serum was calculated based on RT-qPCR results using the prepared standard curve. Thermal cycling was performed in the following conditions: 95 °C for 3 min; 40 cycles of 95 °C for 5 s; and 60 °C for 30 s.

### 2.7. Detection of PRRSV Antibodies

PRRSV-specific antibody detection in serum samples harvested at 0, 3, 6, 9, 12, and 14 dpi was performed with a commercial ELISA kit (IDEXX HerdChek ELISA, Inc., Westbrook, ME, USA). S/P < 0.4 is considered the threshold for seronegativity.

### 2.8. Statistical Analysis

All measured values in this study were expressed as the mean ± standard deviations (SDs), and statistical differences were analyzed by two-way ANOVA, followed by Tukey’s multiple comparisons test using GraphPad Prism software (version 10, San Diego, CA, USA), and a *p*-value < 0.05 was considered statistically significant.

## 3. Results

### 3.1. Isolation and Identification of PRRSV SCMS2025

**Figure 1:**
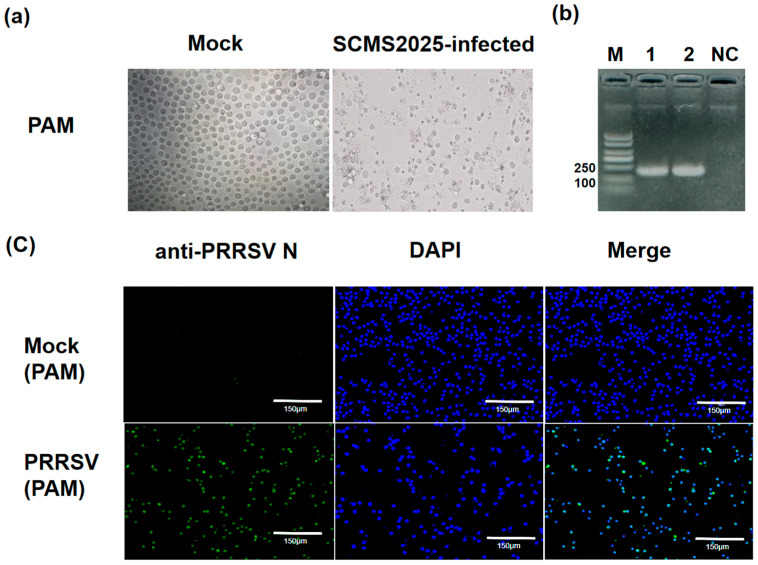
Viral isolation and characterization of SCMS2025. (**a**) PAMs were inoculated with PRRSV SCMS2025, as shown at 60 h post infection. (**b**) Amplification signals with 240 bp were detected in all PAM cells infected with the serum samples using RT-PCR. M, DNA Marker (D2000); 1 and 2, PRRSV SCMS2025 infected cells; and NC, negative control cells. (**c**) PAM cells were infected with seven passage viral cultures after 60 h and examined by IFA with the anti-N PRRSV monoclonal antibody. Cell nuclei are stained with DAPI. Scale bar = 150 μm.

To isolate the virus, positive lung and serum samples of PRRSV were inoculated into the PAMs. Subsequently, obvious CPEs were observed in the inoculated PAM cell wells. Many cells exhibited deformation, detachment, and lysis at 60 h post-inoculation (hpi) (Figure 1a). After seven passages of the virus, two isolates were obtained and named SCMS2025. The presence of PRRSV in the PAMs was further confirmed by RT-PCR (Figure 1b). Furthermore, an immunofluorescence assay was used to visualize the presence of the N protein of SCMS2025 in the infected cells (Figure 1c). After 60 hpi, inoculated PAMs showed specific fluorescence, while the mock-infected cells displayed no observed fluorescence. These results indicated that SCMS2025 isolate could replicate and propagate in PAMs. The TCID_50_ value of the SCMS2025 isolate was 1 × 10^5.0^/mL using PAM cells.

### 3.2. Genomic Information and Phylogenetic Analysis

The complete genome of the SCMS2025 strain was 14,877 bp in length. The genomic sequences of the new isolate are stored in the GenBank. The accession number is PX986904. To identify the genomic characteristics of the isolate, eight representative strains (VR-2332, RespPRRS MLV, JXA1, JXA1-P80, TJbd14-1, TJ, NADC30, and CHsx1401) were selected from the PRRSV-2 genotype to perform nucleotide sequence homology analysis. Whole-genome comparative analysis revealed that the PRRSV strain SCMS2025 shared the highest nucleotide (nt) identity of 87.6% with strains in lineage 5 (VR-2332 and RespPRRS MLV), followed by an nt identity of 86.7–86.9% with lineage 1 strains (NADC30 and CHsx1401). In contrast, the lowest nt identity range of 83.3–85.1% was observed between SCMS2025 and lineage 8 strains (JXA1, JXA1-P80, TJbd14-1 MLV-like, and TJ) (Table 1). The ORF1b and Nsp3–11 regions of SCMS2025 exhibited an nt identity of 96.5–99.3% with RespPRRS MLV (lineage 5) strains, which was higher than other reference strains. Moreover, the ORF1a, Nsp2, ORF2a, ORF2b, and ORF3–7 regions of SCMS2025 shared the highest nt identity of 84.5–95.0% with NADC30-like (lineage 1) strains. In contrast, the Nsp1 and Nsp12 regions displayed 91.6–93.7% nt similarity with JXA1-like (lineage 8) strains, exceeding the similarities detected with all other tested strains. Notably, Nsp1 exhibited nt similarity levels of 91.6% to the TJbd14-1 MLV-like strain, a value higher than that with its parental TJ strain at 91.1%. Additionally, ORF1b, Nsp3, Nsp5, and Nsp7 regions displayed an nt identity of 96.5–98.3% with RespPRRS MLV (lineage 5), which is a higher percentage than that with its parental VR-2332 strain (nt 96.4–98.2%). The above data suggest that the SCMS2025 isolate may have undergone an MLV-like vaccine chimeric recombination event.

### 3.3. Phylogenetic Analysis

**Figure 2:**
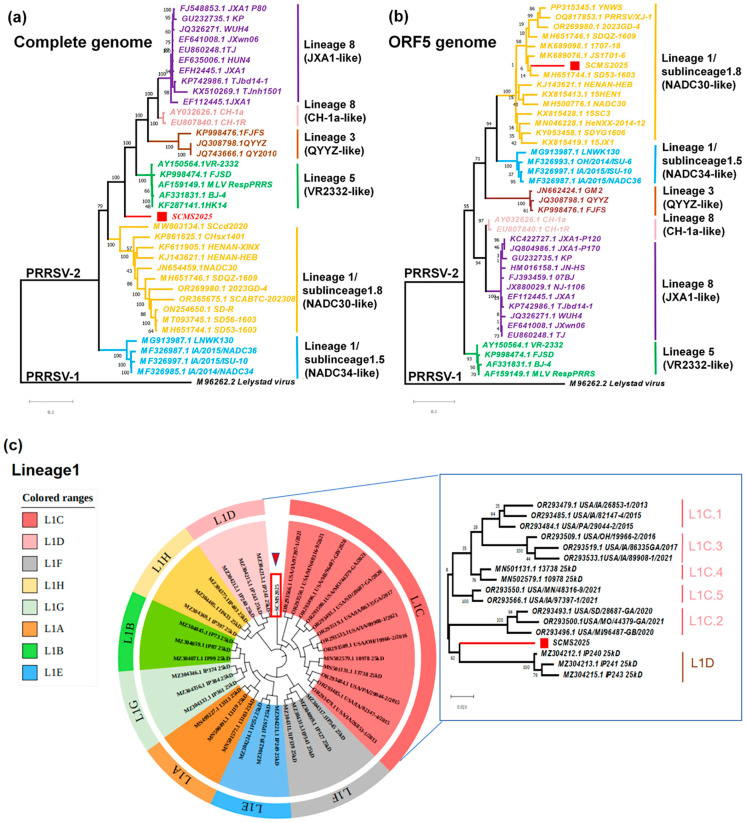
Phylogenetic analysis of SCMS2025. (**a**) Phylogenetic tree based on the whole genome. (**b**) Phylogenetic tree based on ORF5 sequence of SCMS2025 virus strain. (**c**) Phylogenetic tree based on ORF5 nucleotides of PRRSV-2 lineage 1 strains, and the “red Square/Triangle” was SCMS2025. The phylogenetic tree was constructed by the Neighbor-joining method in the MEGA-X software, and the bootstrap values of 1000 replicates were calculated.

The sequenced ORF5 fragments were spliced, and the phylogenetic tree was constructed using MEGA-X software (Figure 2). There are mainly four lineages of PRRSV-2, VR-2332-like (L5A), NADC30-/NADC34-like (L1C), QYYZ-like (L3), and JXA1-/CH-1a-like (L8E), based on the classification of the predominant PRRSV-2 strains in China. Phylogenetic analysis of the complete genome indicated that the present isolate forms a separate branch and clusters between L5 and L1 (Figure 2a). In addition, based on ORF5 gene typing, SCMS2025 belongs to L1 (Figure 2b). According to the latest global classification of L1 lineage, the lineage can be further divided into sublineages L1A–L1H, among which, L1C can be subdivided into L1.1C–L1.5C, and SCMS2025 is located between the L1C and LD lineages (Figure 2c and Supplementary Figure S1). These results collectively indicate that SCMS2025 is likely a recombinant strain.

### 3.4. Amino Acid Analysis of Nsp2

**Figure 3:**
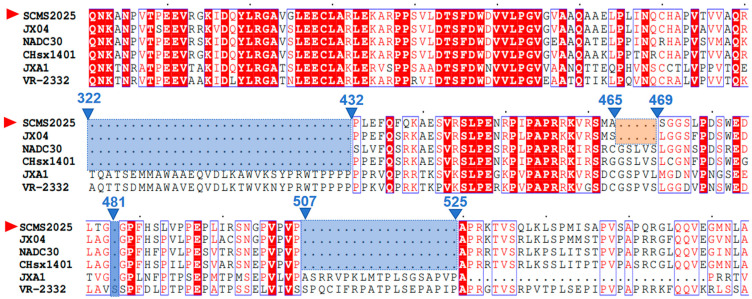
Multiple amino acid sequence alignments of NSP2. Four discontinuous amino acid deletions at positions 322–432, 481, and 507–525 (blue regions) in NSP2 of SCMS2025 and NADC30-like strains. Extra 5-aa deletion at positions 465–469 (orange regions). Red triangle indicates the SCMS2025 isolate; Blue triangle indicates the position of the aa-deletion site.

To confirm the important amino acid characteristics in the PRRSV isolate SCMS2025, the strain was compared with those of other PRRSV reference strains (Figure 3). The red-marked regions represent highly conserved amino acid areas, whereas the unmarked regions indicate differences in amino acids. The SCMS2025 isolate has non-continuous amino acid deletions (111 + 1 + 19-aa), which is typical of the L1 NADC30 deletion: 111-aa deletion at positions 322–432, 1-aa deletion at position 481, and a 19-aa deletion pattern at positions 507–525. It is noteworthy that SCMS2025 contained an extra 5-aa deletion at positions 465–469, exhibiting the same aa deletion as the JX04 stain.

### 3.5. Recombination Analysis

**Figure 4:**
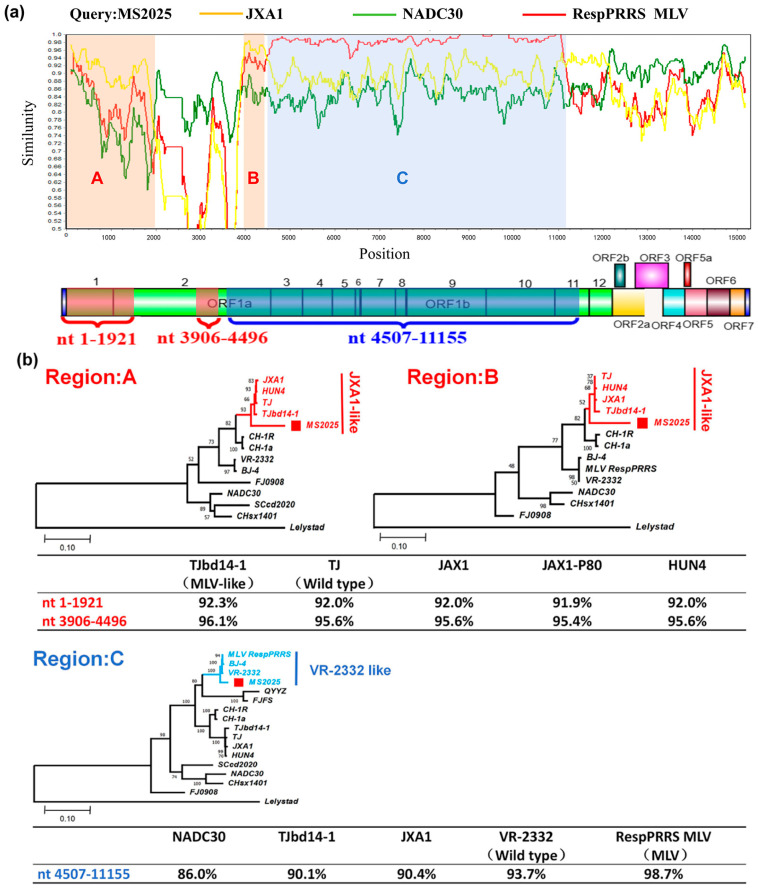
Genome recombination analysis of SCMS2025 isolate. (**a**) The *y*-axis indicates the percentage similarity between the query sequence (SCMS2025) and three representative sequences. Genome scale similarity comparisons of SCMS2025 with JXA1 (yellow), NADC30 (green), and RespPRRS MLV (red). There are three color regions in total. The two light red regions are JXA1, and one light blue region belongs to RespPRRS MLV. (**b**) Phylogenetic trees and nucleotide identity based on each recombinant fragment (regions A–C) of SCMS2025 were constructed to confirm the accuracy of recombination events. Regions A and B indicate the nucleotide sequences of red shadow; region C indicates the nucleotide sequences of light blue shadow. The SCMS2025 isolate in this study is labeled with “Square”.

Gene recombination analysis of the full-genome sequences of the newly isolated strain and reference strains was performed using SimPlot and RDP4 software. The SCMS2025 strain was observed with significant evidence (at least six methods in the RDP4 program) of three recombination regions (Supplementary Table S2). Three recombinant regions (A, B, and C) were identified among JXA1, NADC30 and RespPRRS MLV strains. For region A, six methods (RDP, Bootscan, MaxChi, Chimaera, 3Seq, and SiScan) detected recombination, with *p*-values of 5.571 × 10^−34^–7.803 × 10^−10^. For region B, six methods (RDP, GENECONV, Bootscan, MaxChi, Chimaera, and 3Seq) supported recombination, with *p*-values of 1.725 × 10^−38^–3.281 × 10^−5^. All seven methods confirmed recombination in region C, with *p*-values ranging from 7.920 × 10^−164^–4.440 × 10^−16^. From the similarity plot analysis, five recombination breakpoints were identified, mapping to ORF1a (nt 1921, 3906, 4496, and 4507) and ORF1b (nt 11155) (Figure 4a). Furthermore, phylogenetic tree construction and homology analysis were performed using the sequences of recombination regions (Figure 4b). The results showed that regions A (nt 1–1921) and B (nt 3906–4496) were classified into the L8 (JXA1-like). These two regions shared the highest nucleotide identity with the MLV-like vaccine strain TJbd14-1, with values of 92.3% and 96.1%, respectively, which were higher than those with its strain TJ isolate at 92.0% and 95.6%. Remarkably, region C (nt 4507–11155) was grouped with L5 (VR-2332-like) strains, and it exhibited a 98.7% nucleotide identity with RespPRRSV MLV, which was significantly higher than that with its parental strain VR-2332 (93.7%). Collectively, the naturally recombinant PRRSV strain SCMS2025 identified in this study was likely generated from the recombination event among NADC30-like, TJbd14-1 MLV-like (L8), and RespPRRS MLV (L5) vaccines. The complex recombination pattern with two MLV-like vaccines is being reported in China, representing a rare recombination pattern.

### 3.6. Experimental Infection of Animals

#### 3.6.1. Clinical Signs

**Figure 5:**
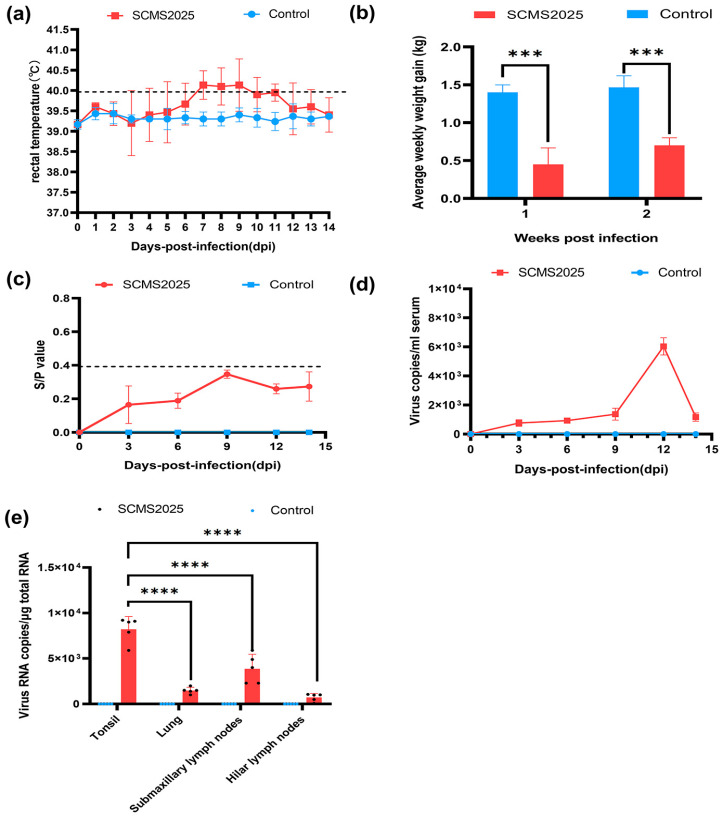
Pathogenicity analysis of the PRRSV isolate SCMS2025 in piglets. (**a**) Rectal temperatures of pigs inoculated with SCMS2025 and control group. The clinical fever cut-off value was set at 40.0 °C. (**b**) Average weekly weight gain of the inoculated pigs during the challenge experiment (1 week *p* = 0.0001; 2 weeks *p* = 0.0005). Asterisk (*) indicates significant differences between the SCMS2025 and inoculated groups (***p < 0.001). (**c**) PRRSV-specific antibodies in serum of challenged pigs at different days post-challenge. S/P > 0.4 was considered as the threshold of serological positivity. (**d**) The PRRSV RNA copy numbers in serum of inoculated pigs at different days post-challenge were detected by RT-qPCR. (**e**) The PRRSV RNA copy numbers in tissues of inoculated pigs post-challenge were detected by RT-qPCR. The tonsils exhibited significantly higher viral loads than lungs, hilar lymph nodes, and submandibular lymph nodes (**** *p* < 0.0001). All measured values in this study were expressed as the mean ± standard deviations (SDs) (*n* = 5 piglets per group), and statistical differences were analyzed by two-way ANOVA followed by Tukey’s multiple comparisons test.

After challenge, SCMS2025-infected pigs developed slight clinical signs within 2–6 dpi, including anorexia, nasal discharge, coughing, and slight diarrhea. At 7–11 dpi, the challenge group began to show severe clinical manifestations such as high fever (>40 °C), hyperpnea, and respiratory distress. Within 12–14 days of viral infection, the clinical symptoms in the piglets in the SCMS2025-challenge group gradually recovered, and the average rectal temperature dropped below 40 °C (Figure 5a). During the challenge period, the SCMS2025-challenge piglets and control pigs showed average weekly gains of 0.45/0.70 kg (1st/2nd week) and 1.40/1.46 kg (1st/2nd week), respectively, with a significant difference (*p* < 0.01) (Figure 5b).

#### 3.6.2. PRRSV-Specific Antibodies and Viral Loads in Serum and Tissues

All serum samples from the SCMS2025-challenge group were collected for PRRSV N protein antibody testing (Figure 5c). Notably, in the SCMS2025-inoculated group, all piglets remained seronegative (S/P < 0.4) during 1–14 dpi. The antibody in the control group also remained negative throughout the experiment. The serum viral RNA copy numbers in the SCMS2025-infected piglets increased slowly at 3 dpi, reached a peak at 12 dpi, and then decreased rapidly at 14 dpi (Figure 5d). Tissue viral load analyses showed that viral levels in the tonsils were significantly higher (*p* < 0.0001) than those in the lungs, hilar lymph nodes, and submandibular lymph nodes (Figure 5e).

#### 3.6.3. Pathological Examination and Immunohistochemistry

**Figure 6:**
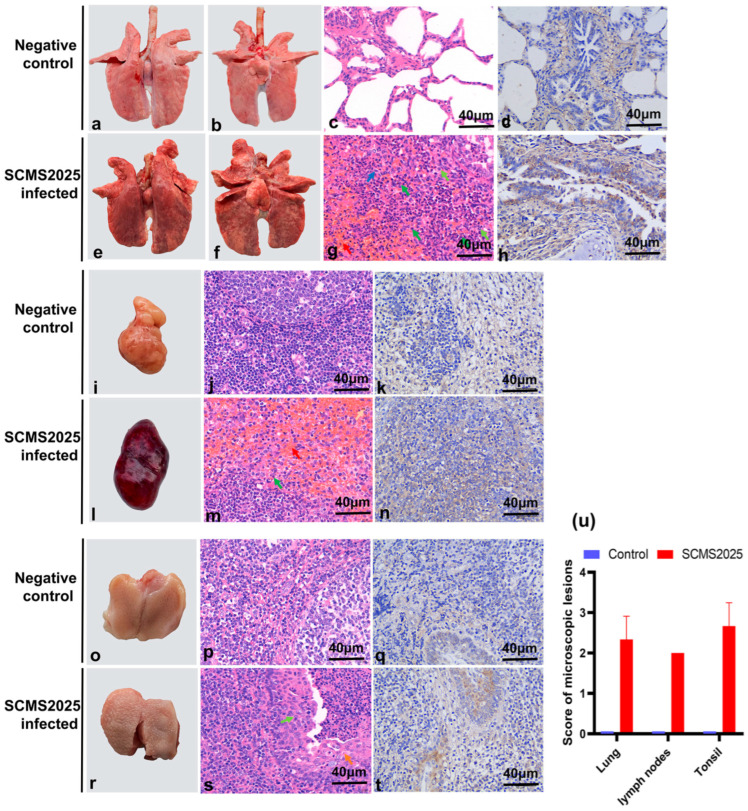
Lung, lymph node, and tonsil lesions observation of the inoculated piglets. (**a**,**b**) No gross lung changes were observed in the negative control pigs. (**e**,**f**) Hemorrhagic pneumonia of lungs with pulmonary consolidation and edema were observed in SCMS2025-inoculated pigs. (**c**) No microscopic lesions were observed in the control piglets. (**g**) Interstitial pneumonia characterized by marked thickening of the alveolar septa, the hyperplasia and necrosis of the alveolar epithelium (light green arrow), inflammation characterized by infiltrating neutrophils (deep green arrow) and fibroblast (blue arrow), and hemorrhage caused by the aggregation of red blood cells in the alveolar cavity and interstitium (red arrow). (**d**) No PRRSV-positive signals were observed in the control pigs. (**h**) PRRSV-specific antigen positive signals mainly distributed in the lungs. (**i**) No gross lymph node changes were observed in the pigs. (**l**) The lymph nodes of pigs inoculated with the SCMS2025 strain exhibited swelling and hemorrhage. (**j**) No microscopic lesions were observed in the control piglets. (**m**) Inflammation characterized by infiltrating neutrophils (green arrow), hemorrhage (red arrow), and pigment deposition (yellow arrow). (**k**) No PRRSV-positive signals were observed in the control pigs. (**n**) Positive PRRSV-specific antigens were detected in the lymph nodes. (**o**) No gross tonsil changes were observed in the SCMS2025-challenge group. (**r**) No changes were observed in the negative group. (**p**) No microscopic lesions were observed in the control piglets. (**s**) The tonsils exhibited pathological damage characterized by degenerative and necrotic crypt epithelial cells (orange arrow) and necrosis (green arrow). (**q**) No PRRSV-positive signals were observed in the negative group. (**t**) Positive PRRSV-specific antigens were detected in the tonsils. (**u**) Pathological severity was scored using a 0–4 scale: normal = 0, mild = 1, moderate = 2, marked = 3, and severe = 4.

All pigs in the SCMS2025-challenge group were euthanized and necropsied. The main histopathological changes were observed in the lungs. These changes included hemorrhage, pulmonary consolidation, edema, and the destruction of the normal alveolar architecture (Figure 6e,f). The hilar lymph nodes showed severe hemorrhage and swelling (Figure 6l). Tonsils in both groups showed no macroscopic pathological lesions (Figure 6o,r). No obvious pathological changes were found in the negative control pigs (Figure 6a,b,i,o).

In the challenge group, HE staining and microscopic histopathological examination revealed characteristic features of interstitial pneumonia, including thickened alveolar septa, inflammatory cell infiltration, alveolar epithelial hyperplasia, and hemorrhage caused by the aggregation of red blood cells in the alveolar cavity and interstitium (Figure 6g). The hilar lymph node sections exhibited predominantly acute inflammatory cell infiltration, hemorrhage, and pigment deposition (Figure 6m). The tonsil showed marked degeneration and necrosis, characterized by cellular swelling, karyopyknosis, or karyorrhexis. In select regions, the epithelial layer of the tonsils lost structural integrity, concurrent with the emergence of proliferative epithelial changes (Figure 6s). None of the tissue samples from the negative group showed pathological changes (Figure 6c,j,p).

IHC staining and microscopic examination revealed that the lungs (Figure 6h), lymph nodes (Figure 6n), and tonsils (Figure 6t) exhibited characteristic brown chromogen labeling, indicating positive immunoreactivity for the PRRSV antigen within the cytoplasm of infected cells. No macroscopic and microscopic pathological changes were detected in the negative group (Figure 6d,k,q). In addition, the ORF5 gene from the lungs, lymph nodes, and tonsils was amplified (primers: 12F/R, Supplementary Table S1) and sequenced to confirm that it was the original virus.

## 4. Discussion

PRRSV has been highly prevalent in pig populations, and the associated clinical disease remains poorly controlled worldwide, causing severe economic losses to the global swine industry. Vaccination is the most effective prevention and control strategy in China [4,24,25]. Since 2009, multiple MLV vaccines, belonging to lineage 8 (JXA1-R, TJM-F92, HuN4-F112, R98, and GDr180) and lineage 5 (RespPRRS PRRS MLV) have been widely used on pig farms for PRRS prevention [26]. Although these MLV vaccines can confer protective efficacy, they still give rise to problems of limited cross-protection against heterologous PRRSV strains [27,28], virulence reversion [29], and recombination with PRRSV field strains [30].

Recombination represents an important mechanism underlying the genetic evolution of PRRSV [31]. To date, numerous studies on recombination events that have occurred between PRRSV field strains and MLV vaccines have been documented in China. Since NADC30-like strains emerged in China in 2013, the viruses have rapidly spread and become the locally dominant virus strain in China currently [32], and they have been reported to have a high incidence of recombination with other heterologous strains (JXA1-like, VR2332-like, QYYZ-like, and NADC34-like) or the MLV/MLV-like strain (JXA1-R, TJM-F92, and RespPRRS PRRS MLV) [13,14,23,33,34,35,36]. Recent PRRSV recombination events outside China, including well-documented cases of vaccine–field-strain recombinants and even vaccine–vaccine recombinants, have been reported in the United States and Europe [37,38,39]. In the present study, we isolated a novel PRRSV strain, SCMS2025, from a pig farm practicing MLV vaccination. Based on the full-length genomic sequences, SCMS2025 is likely a recombinant strain among NADC30-like (L1), a RespPRRS PRRS MLV vaccine (L5), and a TJbd14-1 MLV-like (L8), which is proposed to be an MLV-like strain evolved from the vaccine virus TJM-F92 [15]. Our results revealed that the NADC30-like SCMS2025 strain has undergone complex recombination events involving two distinct MLV-like strains, which are reported in China, representing a rare recombination pattern.

Recently, the accidental usage of two distinct MLVs in the pig herd has been reported to have led to the emergence of a recombinant strain in the United States in 2025. The clinical presentation of the newly emerged PRRSV strain showed mild pathogenicity and did not cause any significant impacts on the downstream pig flow [40]. Similarly, the SCMS2025 strain, which originated from two MLV-like vaccines in China, could cause low mortality and transient clinical disease in this 14-day weaned-piglet model, indicating that SCMS2025 is a low pathogenic strain. However, it must be emphatically acknowledged that the weight gain of the SCMS2025-infected group dropped drastically to 0.45 kg/week. This indicates that, although SCMS2025 exhibits low pathogenicity to piglets based on survival rate, it can still cause severe production losses and considerable economic damage to commercial pig herds. Several potential reasons were considered for the severe growth retardation despite mild pathological changes. Clinically, PRRSV-induced high fever and respiratory distress caused severe anorexia, directly reducing feed intake. Immunologically, robust viral replication in the tonsils (Figure 5e) triggered severe immune stress, metabolically redirecting the host’s energy from growth to systemic inflammation.

Previous studies revealed that the recombination pattern was responsible for the variations in pathogenicity of PRRSV strains [41]. PRRSV NADC30 is a moderately virulent strain first identified in 2008 in the United States [42]. Based on clinical manifestations, some PRRSV recombination events between NADC30-like and the Chinese HP-PRRSV strains have created very aggressive strains, an example being the JL580 that emerged in Jilin province and the FJ1402 in Fujian province in 2014, threatening local swine production [43,44]. Nevertheless, the recombinant PRRSVs derived from NADC30-like and low pathogenic strains exhibit mild pathogenicity (e.g., CHsx1401) [45]. Previous studies demonstrated that a laboratory-created wild-type and MLV vaccine recombinant PRRSV has less clinical symptoms compared to the wild-type strain but is not as attenuated as the MLV [40]. As evaluated in animal experiments, the SCMS2025 recombinant strain showed low pathogenicity in piglets. However, our study lacks parallel in vivo comparisons with reference strains (e.g., non-recombinant NADC30-like, MLV, or HP-PRRSV strains). Although we hypothesize that MLV-like integrations contribute to SCMS2025’s low lethality, we cannot definitively attribute the clinical phenotype to the MLV-like genomic regions or quantify the attenuation. Future studies incorporating reference strains and reverse genetics are required to elucidate the precise molecular mechanisms underlying the pathogenicity of this complex recombinant strain.

Of note, all piglets challenged with the SCMS2025 strain remained seronegative for PRRSV-specific antibodies (S/P < 0.4) throughout the entire 14-day observation period. Typically, specific antibodies against the majority of PRRSV-2 strains can be detected, starting from 10 to 14 dpi [35,46,47,48]. The absence of seroconversion within 14 dpi in our study suggests a delayed humoral immune response, which may primarily be attributed to the relatively short observation period. Therefore, future studies should extend the follow-up period to 21 or 28 days and incorporate measurements for neutralizing antibodies, cytokines, and cellular immunity to fully evaluate the host immune response. On the other hand, a low viral replication level in the host may not provide sufficient antigenic stimulation to trigger a rapid humoral response. Additionally, given the complex recombinant nature of SCMS2025 (incorporating sequences from an NADC30-like isolate and two MLVs), an antigenic mismatch between the challenge strain and the antigens coated in the commercial ELISA kit may affect assay sensitivity, leading to delayed or undetected seroconversion. Furthermore, PRRSV is well known for its ability to modulate host immune responses and induce immunosuppression, thereby delaying the onset of robust adaptive immunity. Previous studies have demonstrated that, compared to highly pathogenic PRRSV (HP-PRRSV) strains, NADC30-like strains can exhibit distinct immunomodulatory properties [49]. Therefore, we hypothesize that the complex recombinant pattern of the SCMS2025 strain (uptaking of nucleotide sequences from a field NADC30-like isolate and two MLVs) may facilitate an enhanced immune evasion mechanism, delaying the activation of B cells and the subsequent production of specific immunoglobulins during the initial phase of infection. Although viremia was detected, the delayed antibody response indicates a complex virus–host interaction. Therefore, whether the SCMS2025 strain can induce distinct immune responses in piglets deserves further study. Finally, the limitation of this study is that the sample size was restricted to five piglets per group due to animal welfare and facility constraints. Although sufficient for observing overall clinical trends, it may limit the statistical power. Future studies with larger sample sizes (n ≥ 6) are warranted.

## 5. Conclusions

A PRRSV strain, SCMS2025, was isolated from the whole blood samples of diseased pigs in Southwestern China in 2025. Genomic recombination analysis revealed that the newly emerged PRRSV isolate was likely generated by multiple recombination events between NADC30-like and two MLV-like strains (RespPRRS MLV and TJbd14-1 MLV-like strains), which are reported in China, representing a rare recombination pattern. Animal experiments demonstrated that SCMS2025 induced transient clinical signs, decreased weight gain, persistent viremia, detectable viral RNA in tissues, and pathological lesions in weaned piglets during the 14-day observation period, accompanied by a delayed antibody response. Our study highlights the importance of developing new strategies for PRRS vaccines.

## Figures and Tables

**Table 1:** Nucleotide identity of SCMS2025 compared with eight PRRSV reference strains.

Nucleotide Identity% (SCMS2025)								
Region	VR-2332 (AY150564)	RespPRRS MLV (AF159149)	JXA1 (EFH2445)	JXAI-P80 (FJ548853)	TJbd14-1 (MLV) (KP742986)	TJ (EU860248)	NADC30 (JN654459)	CHsx1401 (KP861925)
	VR-2332-like (Lineage 5)		JXA1-like (Lineage 8)				NADC30-like (Lineage 1)	
Complete genome	**87.6**	**87.6**	85.1	84.9	83.3	85.1	86.7	86.9
ORF1a	83.3	83.1	80.3	80.2	76.7	80.2	**84.5**	84.3
ORF1b	96.4	**96.5**	91.6	91.5	91.3	91.6	86.5	87.5
NSP1	84.3	84.2	91.1	91.2	**91.6**	91.1	81.1	79.5
NSP2	65.5	65.4	64.0	63.7	54.6	63.8	**84.8**	84.6
NSP3	97.0	**97.2**	91.1	91.1	91.0	91.1	84.6	84.9
NSP4	**98.5**	**98.5**	88.9	88.9	88.9	88.9	84.5	85.7
NSP5	90.6	**97.1**	88.2	88.4	88.2	88.2	87.1	86.5
NSP6	**97.9**	**97.9**	89.6	87.5	89.6	89.6	85.4	85.4
NSP7	98.2	**98.3**	89.3	89.0	88.9	89.0	84.8	85.4
NSP8	**99.3**	**99.3**	97.1	97.8	97.8	97.8	91.2	92.6
NSP9	**99.1**	**99.1**	91.8	91.8	91.7	91.7	87.3	88.3
NSP10	**99.0**	**99.0**	89.5	89.5	89.2	89.6	85.2	85.8
NSP11	**99.0**	**99.0**	89.5	89.5	89.2	89.6	85.2	85.8
NSP12	87.1	87.1	**93.7**	93.2	93.5	93.7	85.8	86.1
ORF2a	86.2	86.0	85.3	84.9	85.3	85.3	91.9	**92.1**
ORF2b	88.2	88.2	88.2	88.7	87.8	88.2	**95.0**	93.7
ORF3	82.4	82.4	82.1	81.7	82.5	82.0	**91.6**	**91.6**
ORF4	87.3	87.3	85.5	84.2	86.8	86.4	**93.3**	92.2
ORF5	84.4	84.4	83.2	83.2	83.2	83.4	89.7	**90.2**
ORF6	88.2	88.0	87.8	87.4	87.4	87.8	**94.3**	94.1
ORF7	88.6	88.6	86.2	86.2	85.7	86.2	**90.8**	90.3

Bolded numbers depict the highest percentage identity.

## Data Availability

The data used to support the findings of this study are included within the article. The PRRSV nucleotide sequence data of this study were submitted to GenBank database (http://www.ncbi.nlm.nih.gov/genbank/, accessed on 16 June 2026) under accession number: PX986904 (03-MAY-2026).

## Supplementary Materials

The following supporting information can be downloaded at: https://www.mdpi.com/article/10.3390/ani16121903/s1, Table S1: Specific primers used to amplify the genome of PRRSV SCMS2025 of in this study. Table S2: Information on recombination events detected in SCMS2025. Figure S1: Phylogenetic tree based on ORF5 nucleotides of PRRSV-2 lineage 1 strains.
