# Supplementary material for: Emergence of a Novel Porcine Reproductive and Respiratory Syndrome Virus 2 Strain Recombined from Two Modified Live Virus-like Strains and Its Pathogenicity for Piglets
Source: Animals (Basel). 2026 Jun 19;16(12):1903. doi: 10.3390/ani16121903 (PMC13295636; doi:10.3390/ani16121903)
Supplement: Supplementary file 1 [file animals-16-01903-s001.zip › Supplementary Material 2.pdf]

Supplementary Material 2

Supplementary Tables

Table S2: Information on recombination events detected in SCMS2025

Recombinant strain	Breakpoints		Parental Sequence		Detection methods (p-value)						
	**Beginning**	**Ending**	**Major**	**Minor**	**RDP**	**GENECONV**	**BootScan**	**MaxChi**	**Chimaera**	**SiScan**	**3Seq**
**SCMS2025**	1	1921	NADC30	JXA1	5.571×10^{-34}	NS	7.054×10^{-34}	6.797×10^{-26}	7.803×10^{-10}	6.664×10^{-26}	8.881×10^{-16}
	3906	4496	NADC30	JXA1	1.725×10^{-38}	1.310×10^{-16}	3.769×10^{-24}	1.287×10^{-10}	3.218×10^{-05}	NS	8.881×10^{-16}
	4507	11155	NADC30	RespPRRS MLV	1.861×10^{-150}	7.920×10^{-164}	4.755×10^{-157}	6.484×10^{-56}	2.111×10^{-42}	5.819×10^{-81}	4.440×10^{-16}
