# Supplementary material for: Emergence of a Novel Porcine Reproductive and Respiratory Syndrome Virus 2 Strain Recombined from Two Modified Live Virus-like Strains and Its Pathogenicity for Piglets
Source: Animals (Basel). 2026 Jun 19;16(12):1903. doi: 10.3390/ani16121903 (PMC13295636; doi:10.3390/ani16121903)

Supplementary Material 3

Supplementary Figure

Figure S1: phylogenetic tree based on ORF5 nucleotides of PRRSV-2 lineage 1 strains

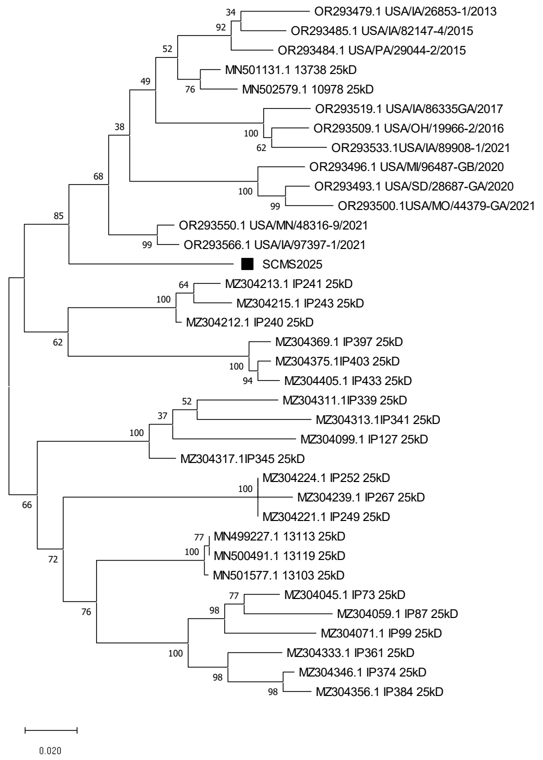

Supplement: Supplementary file 1 [file animals-16-01903-s001.zip › Supplementary Material 3.pdf]
