# Supplementary material for: Emergence of a Novel Porcine Reproductive and Respiratory Syndrome Virus 2 Strain Recombined from Two Modified Live Virus-like Strains and Its Pathogenicity for Piglets
Source: Animals (Basel). 2026 Jun 19;16(12):1903. doi: 10.3390/ani16121903 (PMC13295636; doi:10.3390/ani16121903)
Supplement: Supplementary file 1 [file animals-16-01903-s001.zip › Supplementary Material 1.pdf]

Supplementary Material 1

Supplementary Tables

Table S1: Specific primers used to amplify the genome of PRRSV SCMS2025 of in this study.

Virus	Primer name [a]	Primers sequence (5´-3´)	Primer position	Length (bp)
1	1F	GCCACGGCATTTGTAT	1-15	518
	1R	CGACCCGCACCATTCT	504-519	
2	2F	TCCCTAACACCTTGCTT	133-147	1500
	2R	ACCACAGTTCCCTTCG	1618-1633	
3	3F	CTATGGTCGCTCATCACG	1380-1397	2065
	3R	ATGGGAAACGAGGCTAAA	3427-3445	
4	4F	CTCGTCTCCCATCTCC	3043-3058	1965
	4R	CACCGCAGTCACATAAA	4992-5008	
5	5F	GGCTACTCCACCACAAACCGTGAAG	4835-4859	1502
	5R	CACCGCAGTCACATAAA	6321-6337	
6	6F	TTGGCTGACATCCTCTG	5195-5211	2241
	6R	CCCGCCCATAACATAG	7421-7436	
7	7F	TGAGGACCGTTTGAATAAG	6587-6605	2273
	7R	GGTGACAGTTTGCCAGTTTT	8841-8860	
8	8F	GATGCCGCACTGAAAG	8220-8235	[illegible]
	8R	ATCCGTCTGGAAACCC	9753-9768	

9	9F	TGAATGGTTTGAAGAGCTCGT	9413-9433	1531
	9R	GTTCTGCCGGGAGTTTAGCAA	10922-10944	
10	10F	GTGTATGACCCACACAGGCAA	10638-10658	661
	10R	TTTGGCACCATAAGGTGTCAA	11278-11298	
11	11F	GCGAGAAGTTGCCGAGTC	10908-10925	2184
	11R	GATAGTGATGTAAACGGGTGT	13070-13090	
12	12F	AAAATCCCTCAATGTCGCAGTGTAAC	12958-12983	1866
	12R	CAGTGTAACTTATTCTCCCT	14805-14824	
13	13F	AGATTATCGCCCAACAGA	14644-14661	233
	13R	TAGGCAAACTAAACTCCACA	14858-14877	

[a] F and R represent forward and reverse primers, respectively.
